# Landscape of Metabolic Fingerprinting for Diagnosis and Risk Stratification of Sepsis

**DOI:** 10.3389/fimmu.2022.883628

**Published:** 2022-05-18

**Authors:** Geng Lu, Jiawei Zhou, Ting Yang, Jin Li, Xinrui Jiang, Wenjun Zhang, Shuangshuang Gu, Jun Wang

**Affiliations:** ^1^ Department of Emergency, Nanjing Drum Tower Hospital, The Affiliated Hospital of Nanjing University Medical School, Nanjing, China; ^2^ Department of Pharmacy, Nanjing Drum Tower Hospital, The Affiliated Hospital of Nanjing University Medical School, Nanjing, China; ^3^ Departments of Laboratory Medicine, Nanjing Drum Tower Hospital, The Affiliated Hospital of Nanjing University Medical School, Nanjing, China

**Keywords:** sepsis, septic shock, biomarkers, metabolomics, risk score

## Abstract

**Background:**

Sepsis and septic shock, a subset of sepsis with higher risk stratification, are hallmarked by high mortality rates and necessitated early and accurate biomarkers.

**Methods:**

Untargeted metabolomic analysis was performed to compare the metabolic features between the sepsis and control systemic inflammatory response syndrome (SIRS) groups in discovery cohort, and potential metabolic biomarkers were selected and quantified using multiple reaction monitoring based target metabolite detection method.

**Results:**

Differentially expressed metabolites including 46 metabolites in positive electrospray ionization (ESI) ion mode, 22 metabolites in negative ESI ion mode, and 4 metabolites with dual mode between sepsis and SIRS were identified and revealed. Metabolites 5-Oxoproline, L-Kynurenine and Leukotriene D4 were selected based on least absolute shrinkage and selection operator regularization logistic regression and differential expressed between sepsis and septic shock group in the training and test cohorts. Respective risk scores for sepsis and septic shock based on a 3-metabolite fingerprint classifier were established to distinguish sepsis from SIRS, septic shock from sepsis. Significant relationship between developed sepsis risk scores, septic shock risk scores and Sequential (sepsis-related) Organ Failure Assessment (SOFA), procalcitonin (PCT) and lactic acid were observed.

**Conclusions:**

Collectively, our findings demonstrated that the characteristics of plasma metabolites not only manifest phenotypic variation in sepsis onset and risk stratification of sepsis but also enable individualized treatment and improve current therapeutic strategies.

## Introduction

Sepsis, which is characterized by life-threatening organ dysfunction caused by dysregulated host response to infection, is a critical clinical status (1, 2). Sepsis is one of the most common critical and causes of death, with more than 31 million cases estimated to be newly diagnosed, and it accounts for 5.3 million deaths per year worldwide (3). Septic shock, a subset of sepsis with higher risk stratification, occurs with a critical reduction in circulatory function, tissue damage and acute failure of multiple organs, and the short-term mortality rate is estimated to be approximately 40% to 70% (3, 4).

Currently, biomarkers that enable early and accurate identification of sepsis and risk stratification may help inform clinical decision-making and potentially assist in formulating appropriate treatment strategies to improve patient management (5). Active case finding has shown that most mild sepsis sufferers may benefit most from prompt effective treatment. Humoral biomarkers, including procalcitonin (PCT) (6), C-reactive protein (CRP) (7), lactate (8) and other dysregulated blood noncoding RNAs (9), are generally employed in clinical practice to help physicians assess sepsis and perform risk stratification. However, few of them have satisfactorily addressed the challenge of distinguishing sepsis from critical illness, and they are susceptible to being influenced by other diseases and clinician experience. More importantly, the intrinsic heterogeneity and its variability at the individual patient level pose significant challenges to discern sepsis and septic shock (10).

Metabolomics has emerged as a promising technology to facilitate the metabolic signatures of disease and the discovery of candidate biomarkers, particularly noninvasive blood biomarkers (11). It is well documented that disorders of hypermetabolism are regarded as challenging, and sepsis is accompanied by metabolic dynamic process, such as a high catabolic state (12), increased mobilization of amino acids (13), and metabolic hyperactivity (14). Consequently, sepsis is likely to be related to an altered profile of blood metabolites, which may reflect its severity and be applied as a diagnostic tool.

Here, we report a high-resolution landscape of metabolic fingerprinting for the diagnosis and risk stratification of sepsis. First, we used untargeted metabolomic profiling and targeted metabolite quantification analysis to conduct a comprehensive analysis of sepsis and septic shock-related plasma metabolites and a 3-metabolite fingerprint classifier including 5-Oxoproline, L-Kynurenine and Leukotriene D4 during the morbidity of sepsis and risk stratification of sepsis. Finally, respective risk scores for sepsis and septic shock based on a 3-metabolite fingerprint classifier were established, and the diagnostic power was evaluated in clinical practice. We propose that the characteristics of plasma metabolites not only manifest phenotypic variation in sepsis onset and risk stratification of sepsis but also enable individualized treatment and improve current therapeutic strategies.

## Materials and Methods

### Chemicals and Reagents

MS-grade water, methanol and acetonitrile were supplied by Thermo Fisher Scientific (Bremen, Germany). 5-Oxoproline, L-Kynurenine and Leukotriene D4 were provided by Aladdin (Shanghai, China). Formic acid (FA) was obtained from Sigma Aldrich (St. Louis, MO, USA).

### Plasma Sample Preparation

This research was performed at the Nanjing Drum Tower Hospital between January 2018 and January 2021, and the ethics committee of Nanjing Drum Tower Hospital (2021-360-02) approved the study, which was conducted according to the Declaration of Helsinki from the World Medical Association (WMA). First diagnosed patient (>18 years-old) consecutively admitted into emergency department with early-onset (< 24 h) sepsis/septic shock or SIRS were enrolled in this study with informed written consent from the patient or their guardian. All the samples were collected from patients before therapy. Sepsis was identified by an increase in the SOFA score (sepsis-related) of 2 points or more, in response to an infection according to the Sepsis-3 definition (15). Septic shock was recognized as persisting hypotension requiring vasopressors to maintain a mean arterial pressure of 65 or more and a serum lactate level greater than 2 mmol/L (18 mg/dL). Patients were treated according to the international guidelines for the management of sepsis and septic shock. The SIRS group were defined as non-septic SIRS patients who met at last two of the four conventional criteria for SIRS but did not meet the diagnostic criteria for sepsis or septic shock (16). The exclusion criteria included the following factors: (1) pregnancy; (2) underlying immunosuppression (malignant tumor, immunosuppressive therapy, organ or drug-induced leukopenia); and (3) endocrine metabolic disease other than diabetes.

**Figure 1:**
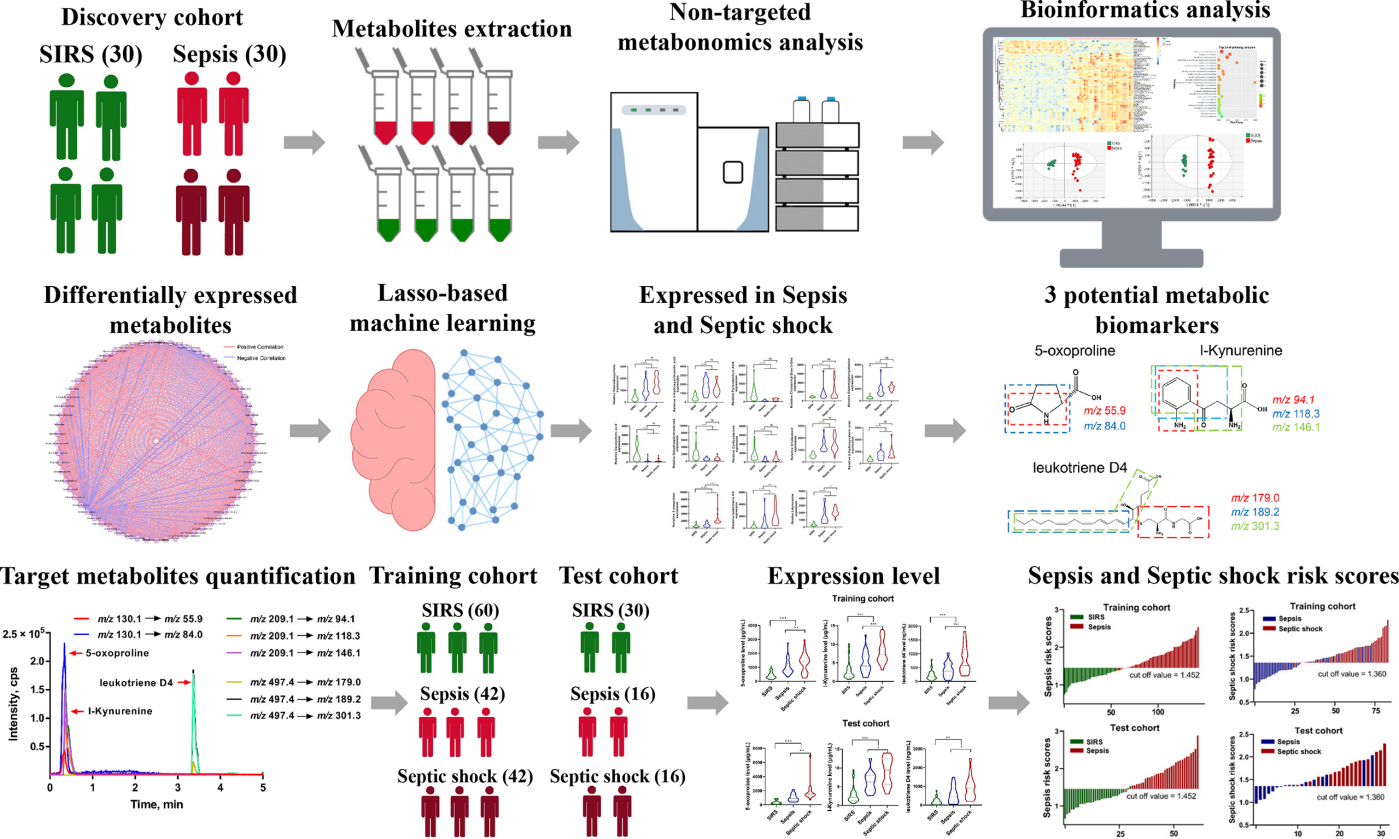
Schematic workflow of Landscape of metabolic fingerprint for diagnosis and risk stratification of sepsis. First, plasma samples from SIRS and sepsis participants were profiled using mass spectrometry-based untargeted metabolomics, and differentially expressed metabolites were identified. Then, least absolute shrinkage and selection operator regularization logistic regression based potential metabolic biomarkers selection was performed, and 3 potential metabolic biomarkers including 5-Oxoproline, L-Kynurenine and Leukotriene D4 were selected. Multiple reaction monitoring (MRM) based target metabolite quantification method was developed and the abundance of 5-Oxoproline, L-Kynurenine and Leukotriene D4 were determined in training and test cohort. Finally, respective risk scores for sepsis and septic shock based on 3-metabolite fingerprint classifier were established and diagnostic power was evaluated in clinical practice.

**Figure 1** presents an outline of this workflow design. According to the diagnostic consensus, 30 sepsis patients, including 15 septic shock patients, and 30 age- and sex-matched SIRS patients (controls), were recruited for the discovery cohort. Then, 116 additional sepsis cases and 90 age- and sex-matched SIRS controls were recruited. From these 116 patients, we randomly selected 84 sepsis participants, including 42 septic shock patients, as the training cohort and the remaining 32 sepsis patients, including 16 septic shock patients, as the test cohort, while 60 and 30 SIRS controls were chosen for the training and test cohorts, respectively.

Plasma was immediately separated from whole blood samples that were collected with EDTA vacuum tubes (BD Vacutainer, Franklin Lakes, NJ, USA) and then stored at -80°C. 100 µL of each plasma sample was slowly lysed, four volumes (400 µL) of precooled methanol were added, and the mixture was vortexed and stored at -80°C for 8 h to allow protein precipitation by centrifugation at 14,000 rpm for 10 min at 4°C. The supernatant was collected, evaporated, reconstituted and used for ultraperformance liquid chromatography plus Q-Exactive Orbitrap tandem mass spectrometry (UPLC-QE-MS) analysis.

### Metabolite Profile and Identification

Plasma extract metabolites were separated by reversed-phase liquid chromatography (RPLC) and analyzed using a Thermo QE HF Orbitrap LC–MS/MS System in both positive and negative electrospray ionization (ESI) ion modes. The MS/MS data acquisition was operated in full MS scan mode and product ion scan with a scan m/z range of 67-1000 Da. Information-dependent acquisition (IDA) and high sensitivity mode were used for secondary mass spectra detection. Quality control (QC) samples that were prepared with aliquots of plasma were injected after every 10 sample injections to monitor system stability. Finally, metabolite identification was completed by distributing the generated MS1/MS2 pairs from public databases, including the MS-DIAL internal database and human metabolome database (http://www.hmdb.ca/). Metabolic features were extracted and aligned with a unique m/z and retention time and then quantified with MS-DIAL v4.24 software.

### Metabolic Network and Pathway Analysis

Pearson’s correlation coefficient of the conversion of signal intensity of significantly varied metabolites by log transformation was calculated, followed by correlation-based metabolic networking analysis (Cytoscape 3.7, Institute of Systems Biology, Seattle). Pathway analysis based on variable metabolites was performed with MetaboAnalyst software (http://www.metaboanalyst.ca).

### Multiple Reaction Monitoring (MRM)-Based Target Metabolite Quantification

For absolute quantitative analysis of potential metabolic markers in the cohort, a SCIEX Exion LC AD system (AB SCIEX, MA, USA) and a QTRAP 5500 mass spectrometer (AB SCIEX, MA, USA) were employed for MRM-based target metabolite quantification. The sample preparation and analysis were performed in basically the same way as in the protocol described previously. The metabolites were separated at a flow rate of 0.3 mL/min with a 10 min gradient [buffer B, 10% (0 min) → 10% (1 min) → 90% (4 min) → 90% (8 min) → 10% (9 min)] using an analytical column (2.7 μm, 30 mm × 3.1 mm; Agilent Technologies, USA), and Q1 and Q3 were both set at unit resolution. Data were collected and analyzed using AB SCIEX Analyst software, sample concentration was quantitated by summation of transitions, and the average metabolite standard intensity was acquired and used to represent the intensity of the target metabolite.

### Clinical Parameters

All participants’ demographic and clinical characteristics, including age, sex, temperature, heart rate, respiratory rate, the SOFA score and comorbidities were obtained after a physical examination at the admission. The enrolled participants underwent blood laboratory tests that included platelet count and total bilirubin, level white blood cell (WBC), total bilirubin, creatinine, CRP, PCT and lactate.

### Statistical Analysis

Data are presented as the mean ± SD. Student’s t test, analysis of variance or the Mann–Whitney U test was performed using SPSS 22.0 software (Chicago, USA). We applied multivariate data analysis, including principal component analysis (PCA) and orthogonal partial least-squares discriminant analysis (OPLS-DA), with SIMCA 14.1 software (Umetrics, Umea, Sweden). Least absolute shrinkage and selection operator regularization logistic regression (LASSO-LR)-based potential metabolic biomarker selection was performed using EmpowerR 3.0 (Shanghai, China). Risk scores of sepsis and septic shock were established using linear and logistic regression patterns. Pearson correlations between risk scores and PCT, lactate and SOFA scores were calculated (GraphPad Prism, USA). Unconditional logistic regression analysis was performed to calculate the odds ratios (ORs) and their 95% confidence intervals (CIs). Receiver operating characteristic (ROC) analysis was also performed to estimate the sensitivity and specificity by the standard method. The Kaplan–Meier method was used to estimate survival rates. A *P* value < 0.05 was deemed highly significant.

## Results

### Demographic and Clinical Characteristics

Detailed demographic and clinical characteristics of the enrolled participants, including the SIRS and sepsis groups, are shown in **Table S1**. Sepsis patients were similar in sex, age, hypertension, diabetes, IHD, COPD, temperature, heart rate, respiratory rate, platelet count and total bilirubin but had higher levels of WBC and PCT in the discovery, training and test cohorts, while the value of MAP was lower in the sepsis group in the training cohort. In addition, sepsis patients had higher SOFA scores in the discovery cohort, creatinine, CRP, lactate and SOFA scores in the training cohort, and creatinine and lactate in the test and cohorts (*P* < 0.05). In addition, no significant differences were found for other demographic and clinical indexes between the sepsis and SIRS groups (*P* > 0.05). Compared with the sepsis group, septic shock sufferers had higher WBC, PCT, lactate and SOFA scores, and no significant differences were found for other demographic and clinical indexes between the sepsis and SIRS groups (*P* > 0.05) (**Table S2**).

### Metabolic Profiling of Sepsis and Identification of Altered Metabolites

**Figure 2:**
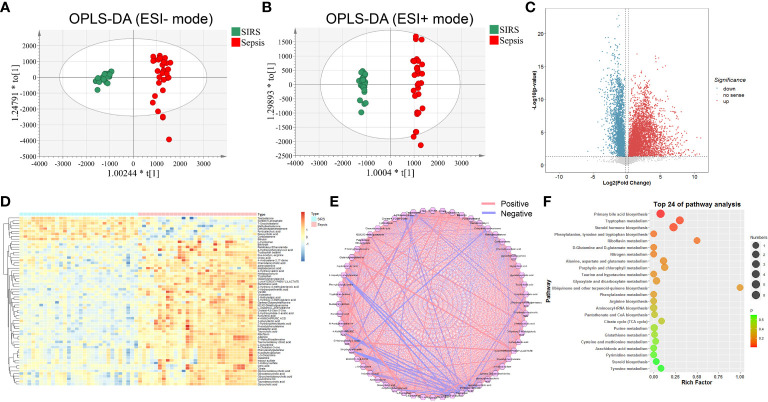
Metabolic profiling of SIRS and sepsis group by mass spectrometry-based untargeted metabolomics analysis. OPLS-DA plots of individual cells of SIRS and sepsis group in negative **(A)** and positive **(B)** ion modes (Negative: Q2(cum) = 0.907; Positive: Q2(cum) = 0.902). **(C)** Volcano plot of significantly increased (red dots)/decreased (blue dots) metabolic features (FC>2,P value<0.05,VIP>1). **(D)** Heatmap of the relative abundance of the metabolites differentially expressed between SIRS and sepsis group. **(E)** Correlation analysis of differential expressed metabolites. **(F)** Metabolic pathway analysis of differential expressed metabolites.

During the discovery process, untargeted metabolomic analysis was performed to compare the metabolic features between the sepsis and control SIRS groups. After quality control, data filtering, and removal of missing values, more than 70,000 metabolic features, including 35480 metabolic features in positive ion mode and 35870 metabolic features in negative ion mode, were consistently identified in plasma samples. Our PCA data showed that QC samples were tightly clustered and were acquired with high stability and reproducibility (**Figures S1A, B**). OPLS-DA analysis was further managed between the sepsis and SIRS groups with Q2(cum) = 0.902 in positive ion mode and Q2(cum) = 0.907 in negative ion mode, suggesting that extensive metabolic changes occur during morbidity (**Figures 2A, B**). Based on the unifying principal rule that a P value under 0.05 with a difference of 2-fold or more and a variable importance projection (VIP) value greater than 1.0, significantly different metabolic features were selected to identify metabolic characteristics and high-confidence metabolites that contribute to the incidence of sepsis. Consequently, significant differences in metabolic features were obtained and visualized by volcano plots (**Figure 2C**). To understand the potential function of sepsis-related metabolites, 64 metabolites, consisting of 46 obtained in positive ESI ion mode, 22 metabolites in negative ESI ion mode, and 4 metabolites with dual mode, were identified and confirmed after inputting the refined significant metabolic features into a public metabolite library (**Table S3**). Differentially expressed metabolites between sepsis and SIRS were revealed in hierarchical clustering analysis (**Figure 2D**). To further investigate the functional groups of metabolites related to sepsis, we utilized correlation analysis on the intensity profiles of the dysregulated metabolites and constructed a metabolic network (**Figure 2E**). According to Kyoto Encyclopedia of Genes and Genomes (KEGG) pathway enrichment analysis, altered metabolites were categorized into 22 metabolic pathways. Based on the enrichment factor and *P* value, primary bile acid biosynthesis, tryptophan metabolism, steroid hormone and others were the most markedly enriched metabolic pathways (**Figure 2F**).

### Potential Metabolic Biomarker Quantification

To further illustrate the metabolic characteristics of sepsis, a LASSO-LR model was employed to select potential metabolic biomarkers. With a grid of values for the normalization parameter lambda, the application model of 13 metabolites suggested the most normalized efficacy among the subjects *via* penalized maximum likelihood (**Figures S2A, B**). Differentially expressed metabolites were assessed to investigate the dynamic variation in metabolites during the progression of sepsis, and interestingly, we found that the metabolites 5-Oxoproline, L-Kynurenine and Leukotriene D4 were elevated with unidirectional behaviors dominating the features and increasing until reaching their peaks in the septic shock process, whereas no significant difference in other metabolites was observed (**Figure S3**).

**Figure 3:**
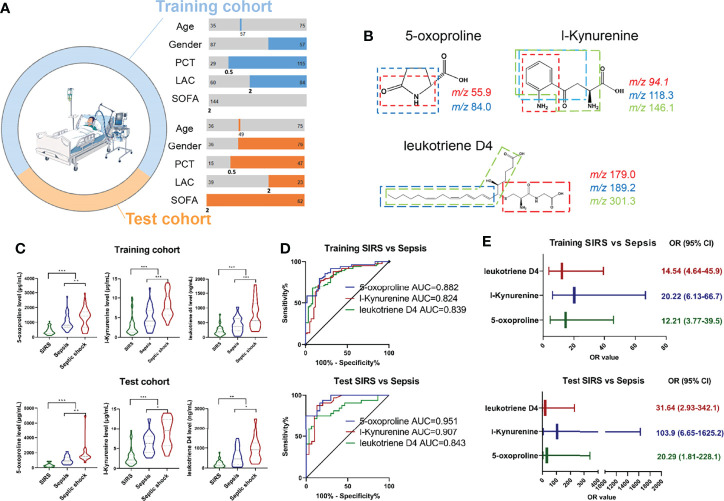
Development of MRM based target metabolite quantification method and detection of the abundance of 3 target metabolites including 5-Oxoproline, L-Kynurenine and Leukotriene D4 in training and test cohort. **(A)** Demographic and clinical characteristics of enrolled cohort. **(B)** Chemical structure and fragmentation of 5-Oxoproline, L-Kynurenine and Leukotriene D4. **(C)** Respective expression of 5-Oxoproline, L-Kynurenine and Leukotriene D4 in the training and test cohorts. **(D)** ROC analysis of 5-Oxoproline, L-Kynurenine and Leukotriene D4 in predicting sepsis in the training and test cohorts. **(E)** Odds ratios of 5-Oxoproline, L-Kynurenine and Leukotriene D4 in predicting sepsis. (^*^, p < 0.05; ^**^, p < 0.01; ^***^, p < 0.001).

Given that 5-Oxoproline, L-Kynurenine and Leukotriene D4 were suggested to be potential diagnostic biomarkers in the discovery cohort, we next focused on the diagnostic utility of 5-Oxoproline, L-Kynurenine and Leukotriene D4 in the training and test cohorts, while the demographic and clinical characteristics are shown in **Table S1** and **Figure 3A**. At least two product ions became a unique signature in combination with the precursor ion for MRM analysis (**Figure 3B**), and MRM transitions of *m/z* 130.1→55.9 and *m/z* 130.1→84.0 for 5-Oxoproline, *m/z* 209.1→94.1, *m/z* 209.1→118.3 and *m/z* 209.1→146.1 for L-Kynurenine, and *m/z* 497.4→179.0, *m/z* 497.4→189.2 and *m/z* 497.4→301.3 for Leukotriene D4 were established, which could then be specific target metabolites for absolute quantitation purposes (**Figure S4A**). The summed value of the target metabolites was used to plot against the calibration curve concentration and sample quantitation (**Figure S4B**). As illustrated in **Figure 3C**, these three metabolites were upregulated in sepsis patients compared with SIRS controls and further upregulated in septic shock patients in the training cohort and test cohort. The areas under the receiver operating characteristic curve (AUCs) of 5-Oxoproline, L-Kynurenine and Leukotriene D4 for the prediction of sepsis were 0.882 (0.828-0.936), 0.824 (0.752-0.897) and 0.839 (0.774-0.904) in the training cohort and 0.951 (0.905-0.997), 0.907 (0.830-0.983) and 0.843 (0.743-0.943) in the test cohort, respectively (**Figure 3D**). More interestingly, the incidence of sepsis was significantly higher among individuals with higher 5-Oxoproline (OR= 14.54, 95% CI=4.61-45.9), L-Kynurenine (OR=20.22, 95% CI=6.13-66.7) and Leukotriene D4 (OR=12.21, 95% CI=3.77-39.5) in the training cohort and 5-Oxoproline (OR=20.29, 95% CI=1.81-228.1), L-Kynurenine (OR=103.92, 95% CI=6.65-1625.2) and Leukotriene D4 (OR=31.64, 95% CI=2.93-342.1) in the test cohort (**Figure 3E**).

### Development of Diagnostic Panel for Sepsis

**Figure 4:**
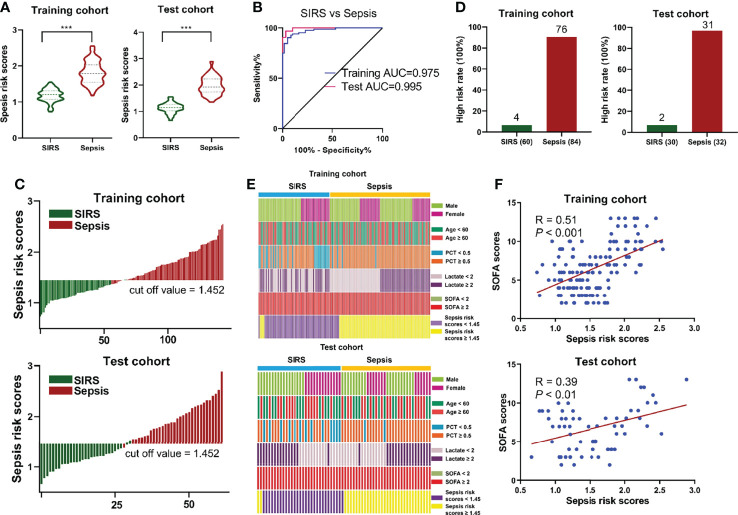
**(A)** Respective values of sepsis risk scores between the sepsis and SIRS groups. The sepsis risk scores were constructed as follows: Ln (sepsis risk scores) = 0.2755 × Ln (5-Oxoproline) + 0.2125× Ln (L-Kynurenine) + 0.1286 × Ln (Leukotriene D4) - 2.2061. **(B)** ROC analysis of sepsis risk scores in predicting sepsis. **(C)** An optimal threshold point value was defined as 1.452, and all participants were divided into low-risk (<cutoff) and high-risk (>cutoff) score groups. **(D)** Analysis of high risk rate between the sepsis and SIRS groups. **(E)** Correlation of sepsis incidence and demographic and clinical characteristics, including age, sex, lactate and SOFA scores, and sepsis risk scores. **(F)** Correlation analysis between sepsis risk scores and SOFA scores. (^***^, p < 0.001).

According to the abundance of three circulating metabolites, the following formula was generated for each individual using a linear and logistic regression model: Ln (sepsis risk scores) = 0.2755 × Ln (5-Oxoproline) + 0.2125× Ln (L-Kynurenine) + 0.1286 × Ln (Leukotriene D4) - 2.2061. The risk scores in the sepsis group were considerably higher than those in the SIRS group in both cohorts (**Figure 4A**). With an AUC of 0.995 (95% CI=0.985-1.000), the sensitivity and specificity of the sepsis risk score were established to be 90.5% and 95.0%, respectively (**Figure 4B**), suggesting the predictive ability of our novel score. An optimal threshold point value was defined as 1.452 using the Youden index in the training cohort. Using the 3-metabolite fingerprint classifier, all participants were divided into low-risk (<cutoff) and high-risk (>cutoff) score groups (**Figure 4C**). When associating the distribution of subjects with the risk score, we found that the subjects with high risk generally had a higher rate of sepsis incidence than those with low risk (**Figure S5**). In addition, the rate of high risk in the sepsis group was predominantly higher than that in the SIRS group (**Figure 4D**). Regarding the incidence of sepsis, no significant association was observed between sepsis incidence and demographic and clinical characteristics, including age, sex, lactate and SOFA scores (**Figure 4E** and **Table S4**). However, a high PCT rate (P < 0.001) and a high sepsis risk score rate (P < 0.001) was associated with sepsis incidence (**Figure 4E** and **Table S3**). Then, the risk score value was shown to be positively correlated with SOFA scores in the training cohort (R = 0.51, P < 0.001) and in the test cohort (R = 0.39, P < 0.01), according to linear correlation analysis (**Figure 4F**). In addition, the risk score value was assessed to be positively correlated with the levels of PCT (R = 0.65, P < 0.001) and lactate (R = 0.46, P < 0.001) in the training cohort and the levels of PCT (R = 0.71, P < 0.001) and lactate (R = 0.56, P < 0.001) in the test cohort (**Figure S6**).

### Correlation Between Target Metabolites and Septic Shock

**Figure 5:**
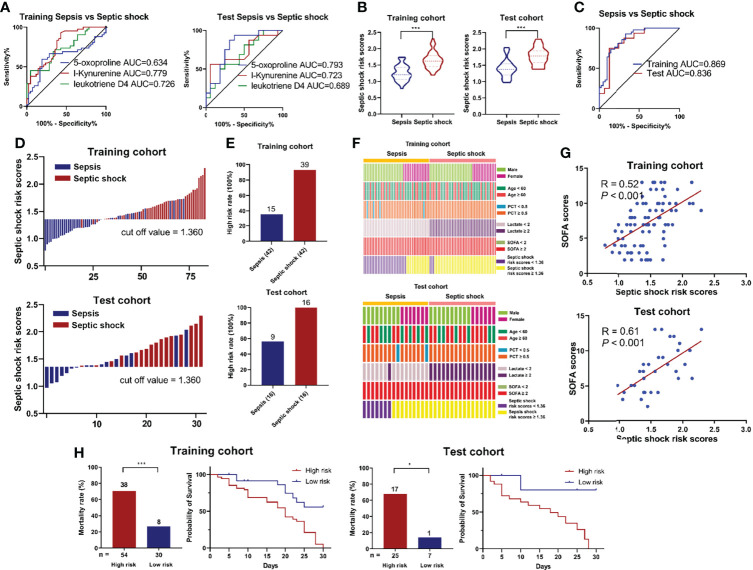
**(A)** ROC analysis of 5-Oxoproline, L-Kynurenine and Leukotriene D4 in predicting septic shock in the training and test cohorts. **(B)** Respective values of septic shock risk scores between the septic shock and sepsis groups. The septic shock risk scores were constructed as follows: Ln (septic shock risk scores) = 0.1095 × Ln (5-Oxoproline) + 0.3791× Ln (L-Kynurenine) + 0.2141 × Ln (Leukotriene D4) - 2.2083. **(C)** ROC analysis of septic shock risk scores in predicting septic shock risk scores. **(D)** An optimal threshold point value was defined as 1.360, and all participants were divided into low-risk (<cutoff) and high-risk (>cutoff) score groups. **(E)** Analysis of high risk rate between the septic shock and sepsis groups. **(F)** Correlation of septic shock incidence and demographic and clinical characteristics, including age, sex, lactate and SOFA scores, and septic shock risk scores. **(G)** Correlation analysis between septic shock risk scores and SOFA scores. **(H)** Survival rates after 30 days of patients in the high and low risk groups. (^*^, p < 0.05; ^***^, p < 0.001).

Given that 5-Oxoproline, L-Kynurenine and Leukotriene D4 were upregulated in the septic shock group (**Figure 3A**), the AUCs for the prediction of septic shock were 0.634 (0.509-0.759), 0.779 (0.681-0.877) and 0.726 (0.619–0.833) in the training cohort and 0.793 (0.632-0.954), 0.723 (0.541-0.904) and 0.688 (0.503–0.872) in the test cohort, respectively (**Figure 5A**). Furthermore, the occurrence of septic shock was significantly higher in subjects with 5-Oxoproline (OR=3.46, 95% CI=1.27-9.45), L-Kynurenine (OR=4.50, 95% CI = 1.53-13.23) and Leukotriene D4 (OR=5.45, 95% CI=18.5-16.09), and similar results indicated that 5-Oxoproline (OR=9.57, 95% CI=1.76-52.14), L-Kynurenine (OR=2.85, 95% CI=0.53-15.21) and Leukotriene D4 (OR = 2.04, 95% CI = 0.38-11.02) were significantly associated with the presence of septic shock (**Figure S7**). According to the abundance of target metabolites, the following equation was derived to estimate septic shock risk scores: Ln (septic shock risk scores) = 0.1095 × Ln (5-Oxoproline) + 0.3791× Ln (L-Kynurenine) + 0.2141 × Ln (Leukotriene D4) - 2.2083. Using the 3-metabolite fingerprint classifier, the septic shock risk scores were shown to be significantly higher than those in the septic shock group in both cohorts (**Figure 5B**). The sensitivity and specificity of the risk score for septic shock were 92.9% and 66.7%, respectively, with an AUC of 0.869 (95% CI=0.793-0.945) in the training cohort and an AUC of 0.836 (95% CI=0.691-0.981) in the test cohort (**Figure 5C**), suggesting its predictive capability. According to the Youden index, the optimal threshold point value was calculated to be 1.360 in the training cohort. Based on the threshold point value (1.360), all subjects were divided into low-risk (<cutoff) and high-risk (>cutoff) score groups (**Figure 5D**). When correlating the status of patients with the risk score, we observed that the participants with high risk generally had a higher proportion of septic shock incidence than those with low risk (**Figure S8**). In addition, the percentage of high-risk patients in the septic shock group was markedly higher than that in the sepsis group (**Figure 5E**). Regarding the occurrence of septic shock, no significant relationship was observed between septic shock incidence and demographic characteristics, including age and sex, or clinical indexes (**Figure 5F** and **Table S5**). However, higher SOFA scores (P = 0.021) and higher risk scores (P < 0.001) were associated with septic shock incidence. Then, the risk score value was assessed to be positively correlated with SOFA scores in the training cohort (R = 0.52, P < 0.001) and in the test cohort (R = 0.61, P < 0.001) (**Figure 5G**), while there were positive correlations between the risk score and the levels of PCT (R = 0.37, P < 0.001) and lactate (R = 0.48, P < 0.001) in the training cohort and the levels of PCT (R = 0.50, P < 0.001) and lactate (R = 0.62, P < 0.001) in the test cohort (**Figure S9**). Besides, the cumulative survival rate for the high risk subgroup was remarkably lower than that for the low risk subgroup (**Figure 5H**).

## Discussion

In the present work, the landscape of metabolic fingerprinting for the diagnosis and risk stratification of sepsis was investigated and identified, while 3 metabolites, 5-Oxoproline, L-Kynurenine and Leukotriene D4, were discovered and confirmed to be gradually upregulated in the sepsis and septic shock groups. The amounts of 5-Oxoproline, L-Kynurenine and Leukotriene D4 compose a metabolic fingerprint classifier that is strongly associated with the diagnosis of sepsis and risk stratification of sepsis. This work emphasizes the possible application of metabolic fingerprinting in predicting sepsis and risk stratification of sepsis.

Sepsis leads to hypermetabolism dysfunction, which is recognized as a major cause of cell metabolism disorders. Due to advanced metabolic demand and inefficiencies in the septic process, several endogenous mechanisms, including a high catabolic state, increased mobilization of amino acids, metabolic hyperactivity and others, support hypermetabolism. Coincidentally, related amino acid pathways, including tryptophan metabolism; phenylalanine, tyrosine and tryptophan biosynthesis; d-glutamine and d-glutamate metabolism; and others, were found to be enriched in the present study (17). Additionally, our enriched pathway bile acid biosynthesis was found to be closely connected to sepsis, while bile acid may possess pro- and anti-inflammatory actions influenced by the time course and level of plasma bile acid during the onset of sepsis (18, 19).

A few studies utilized metabolomics methods to identify biomarkers for sepsis (20, 21). However, these studies were conducted with small sample sizes, and consequently, the results were inconclusive. Additionally, quantitative information on target metabolites in plasma or serum is usually missing in recent studies, whereas quantification in clinical practice is normally performed for marker development and therapeutic target validation. A series of differentially expressed metabolites were identified to unambiguously distinguish sepsis from non-septic SIRS, suggesting that these metabolites might predict the occurrence of sepsis. Therefore, effective biomarkers that reflect the incidence and progression of sepsis were further identified and selected to construct a prediction model.

5-Oxoproline, also named pyroglutamic acid, is a byproduct of disordered glutathione metabolism and has been suggested to be a marker of glutathione status (22). In other research investigating metabolism, 5-Oxoproline was described as a metabolic marker in ischemic stroke (23) and pathological damage of the lung in tuberculosis patients (24) and was closely associated with fulminant type 1 diabetes (25) and colorectal cancer (26). Other evidence has revealed that the accumulation of 5-Oxoproline induces inflammation and impairs antioxidant defenses (27, 28), providing insight into the potential role that oxidative stress plays in the development of sepsis (29). Consistently, 5-Oxoproline has been previously shown to be enriched in sepsis caused by *Pseudomonas aeruginosa* during burn infection (30). Furthermore, accumulating evidence suggests that kynurenine acts as a novel endothelium-derived relaxation factor produced during inflammation (31) and that dysregulation of kynurenine metabolism is related to the release of proinflammatory cytokines in neuropsychiatric diseases (32), cancers (33) and cardiovascular disease (34). Consistently, plasma kynurenine has been shown to be correlated with the development of sepsis and sepsis-related disease (35, 36), suggesting a close relationship between the level of plasma kynurenine and sepsis and sepsis progression. Leukotriene D4, an important cysteinyl leukotriene (cysLT), mainly involved with proinflammatory mediators that initiate inflammation and mount adaptive immune responses for host defense (37). The inflammatory response is a central element of sepsis, and sepsis occurs when the initial appropriate host response to an infection is enhanced and then becomes aberrant. Taken together, our data suggest that the inflammatory response plays a crucial role in sepsis and septic shock and that proinflammatory-related circulating metabolites may also mediate the occurrence and development of sepsis.

In conclusion, combined with untargeted metabolomics, MRM-based target metabolite quantification revealed a metabolic classifier of sepsis and risk stratification of sepsis, with the discovery and validation of specific discriminant metabolites. As a proof of principle, we also revealed that the temporal abundance of metabolic features could be applied to predict the incidence of sepsis and septic shock with high efficiency in a multiplex cohort. Our study further suggested that use of the clinical tools combined with the newly developed risk score according to the 3-metabolite fingerprint classifier in plasma are promising for the diagnosis of sepsis and predication septic shock. Nevertheless, our work demonstrated that the plasma metabolites not only manifest phenotypic variation at sepsis onset and risk stratification of sepsis at an early stage but also enable individualized treatment and improve current therapeutic strategies in an equitable manner.

## Data Availability Statement

The original contributions presented in the study are included in the article/**Supplementary Material**. Further inquiries can be directed to the corresponding authors.

## Ethics Statement

The studies involving human participants were reviewed and approved by The ethics committee of Nanjing Drum Tower Hospital. The patients/participants provided their written informed consent to participate in this study.

## Author Contributions

JW and SG conceived of and designed the experiments, GL and JZ and TY performed the experiments, JL and XJ and WZ. analyzed the data and prepared the figure, SG wrote the manuscript. All authors reviewed the manuscript.

## Conflict of Interest

The authors declare that the research was conducted in the absence of any commercial or financial relationships that could be construed as a potential conflict of interest.

## Publisher’s Note

All claims expressed in this article are solely those of the authors and do not necessarily represent those of their affiliated organizations, or those of the publisher, the editors and the reviewers. Any product that may be evaluated in this article, or claim that may be made by its manufacturer, is not guaranteed or endorsed by the publisher.
