# Supplementary material for: Landscape of Metabolic Fingerprinting for Diagnosis and Risk Stratification of Sepsis
Source: Front Immunol. 2022 May 18;13:883628. doi: 10.3389/fimmu.2022.883628 (PMC9159301; doi:10.3389/fimmu.2022.883628)
Supplement: Supplementary file 1 [file DataSheet_1.docx]

Supplemental Information

**Landscape of metabolic fingerprinting for** **diagnosis and** **risk stratification of sepsis**

Geng Lu^a,#^, Jiawei Zhou^a,#^, Ting Yang^b,#^, Jin Li^a^, Xinrui Jiang^a^, Wenjun Zhang^c^, Shuangshuang Gu^a,*^, Jun Wang^a,*^

^a^Department of Emergency, Nanjing Drum Tower Hospital, The Affiliated Hospital of Nanjing University Medical School, Nanjing, Jiangsu 210008, P.R. China

^b^Department of Pharmacy, Nanjing Drum Tower Hospital, The Affiliated Hospital of Nanjing University Medical School, Nanjing, Jiangsu 210008, P.R. China

^c^Departments of Laboratory Medicine, Nanjing Drum Tower Hospital, The Affiliated Hospital of Nanjing University Medical School, Nanjing, Jiangsu 210008, P.R. China

^#^These authors contributed equally to this work

*Correspondence: Dr Jun Wang, Department of Emergency, Nanjing Drum Tower Hospital, The Affiliated Hospital of Nanjing University Medical School, Nanjing, Jiangsu 210008, P.R. China. Email: wjgaogou@aliyun.com

Dr Shuang-shuang Gu, Department of Emergency, Nanjing Drum Tower Hospital, The Affiliated Hospital of Nanjing University Medical School, Nanjing, Jiangsu 210008, P.R. China. Email: guss2926@njglyy.com

**Table of Contents**

**Table S1.** Detailed Demographic and clinical characteristics of the enrolled participant including SIRS and sepsis group.

**Table S2.** Detailed Demographic and clinical characteristics of the enrolled sepsis and septic shock patients.

**Table S3.** Differential metabolites identified from metabolomics profiling between SIRS and sepsis group.

**Table S4.** Correlation of demographic and clinical characteristics and sepsis risk scores.

**Table S5.** Correlation of demographic and clinical characteristics and septic shock risk scores.

**Figure S1.** Graphical representations of PCA results obtained in negative ion mode **(A)** and positive ion mode **(B)**.

**Figure S2.** Potential metabolic biomarkers selection using the least absolute shrinkage and selection operator regularization (LASSO-LR) model. **(A)** Dotted vertical lines were drawn at the optimal values with Lambda (log), by using the minimum criteria and the 1 standard error of the minimum criteria (the 1-SE criteria). **(B)** OPLS-DA score plot of the analysis using selected metabolite.

**Figure S3.** Respective expression of 5-Oxoproline, L-Kynurenine and Leukotriene D4 between sepsis and septic shock in the training and test cohorts.

**Figure S4.** Development of MRM based target metabolite quantification method **(A)** and standard curve of 5-Oxoproline, L-Kynurenine and Leukotriene D4 **(B)**.

**Figure S5.** Investigation of sepsis incidence rate in high sepsis risk score and low sepsis risk score group which were defined based on a 3-metabolite fingerprint classifier.

**Figure S6.** Correlation analysis between sepsis risk scores and PCT **(A)**, lactate **(B)**.

**Figure S7.** Odds ratios of 5-Oxoproline, L-Kynurenine and Leukotriene D4 in predicting septic shock.

**Figure S8.** Investigation of septic shock incidence rate in high septic shock risk score and low septic shock risk score group which were defined based on a 3-metabolite fingerprint classifier.

**Figure S9.** Correlation analysis between septic shock risk scores and PCT **(A)**, lactate **(B)**.

**
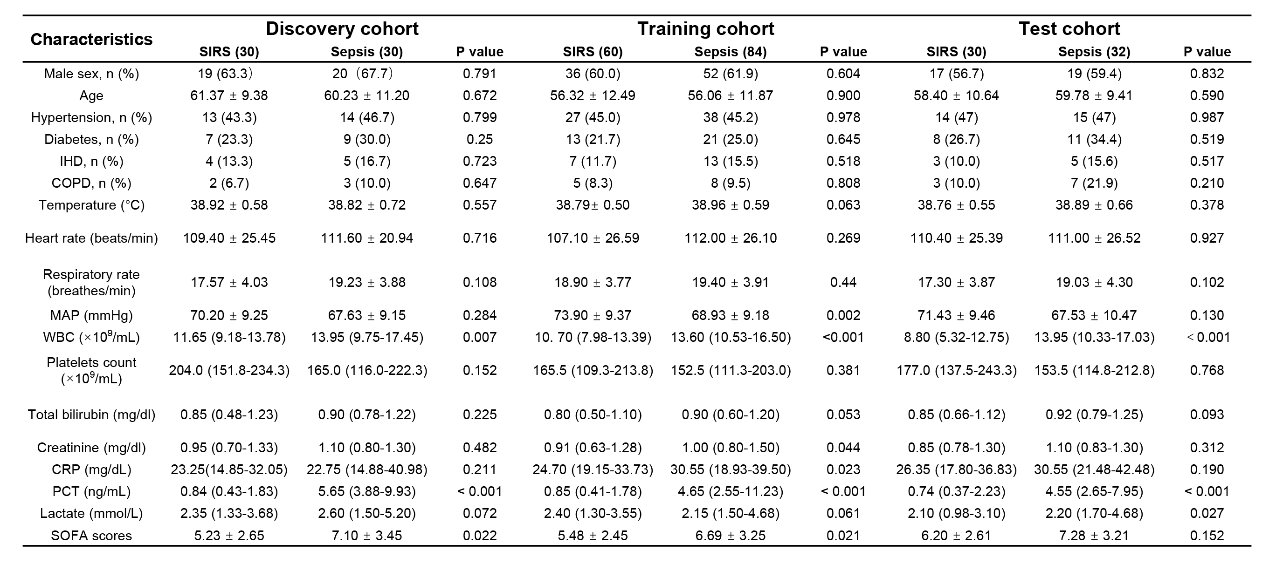
**

**Table S1.** Detailed Demographic and clinical characteristics of the enrolled participant including SIRS and sepsis group.


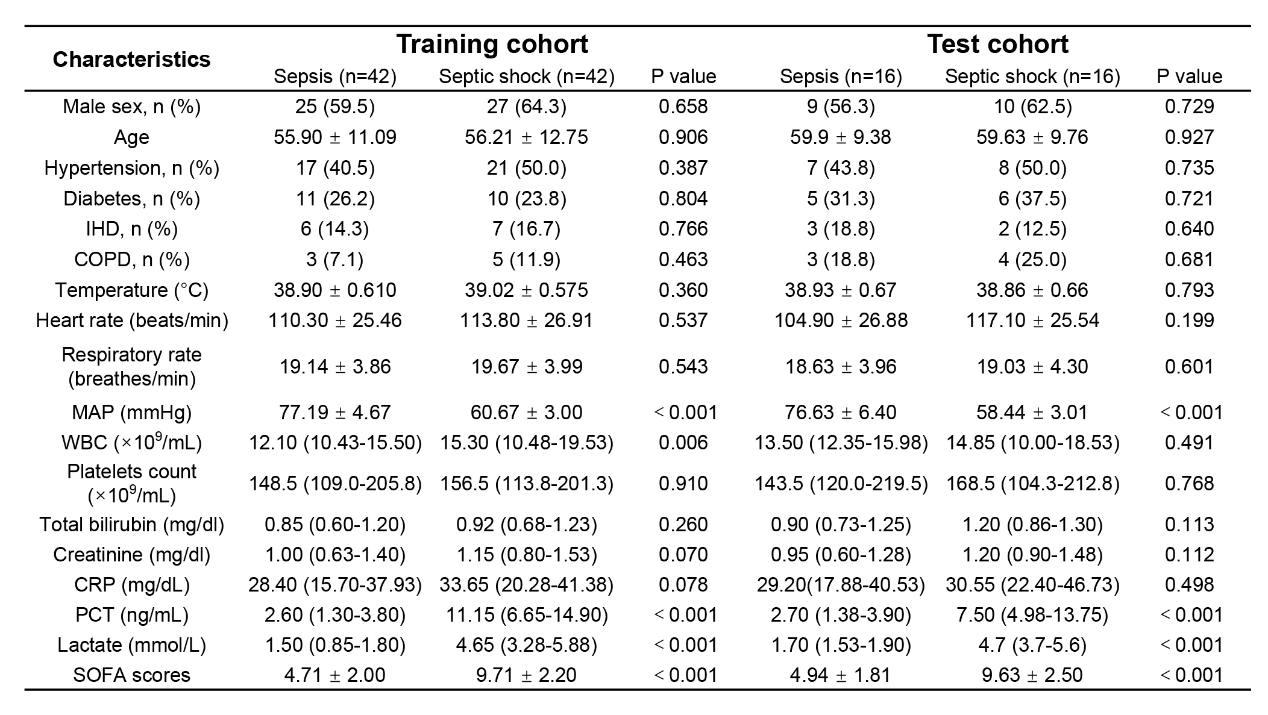


**Table S2.** Detailed Demographic and clinical characteristics of the enrolled sepsis and septic shock patients.

**
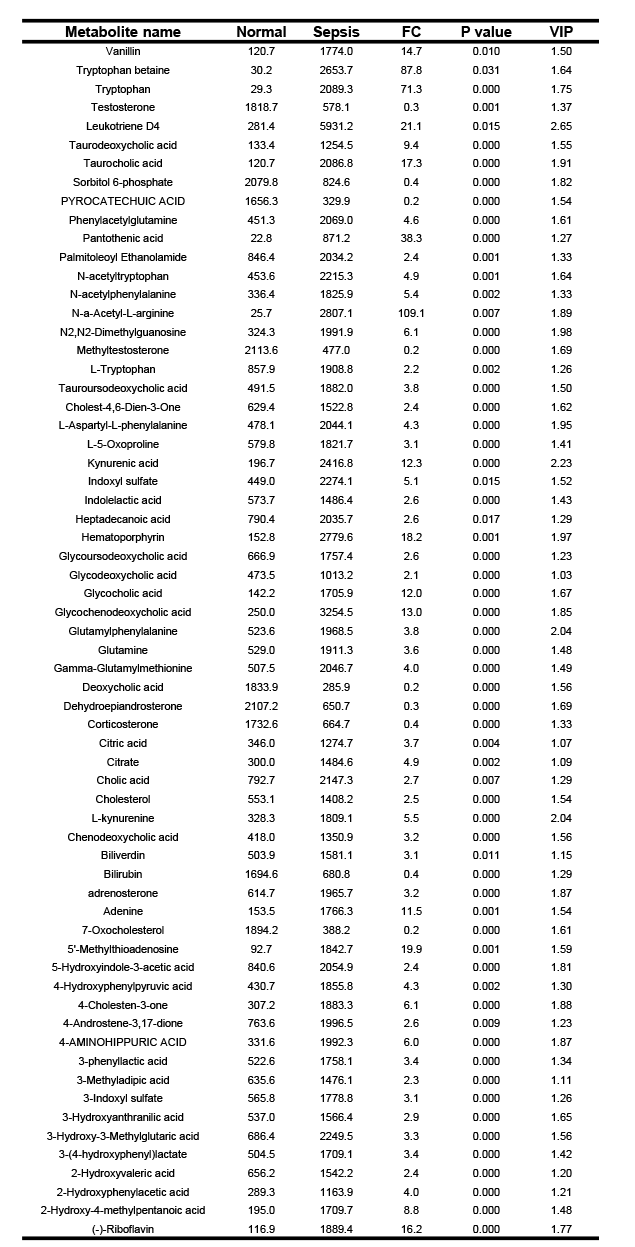
**

**Table S3.** Differential metabolites identified from metabolomics profiling between SIRS and sepsis group.

**
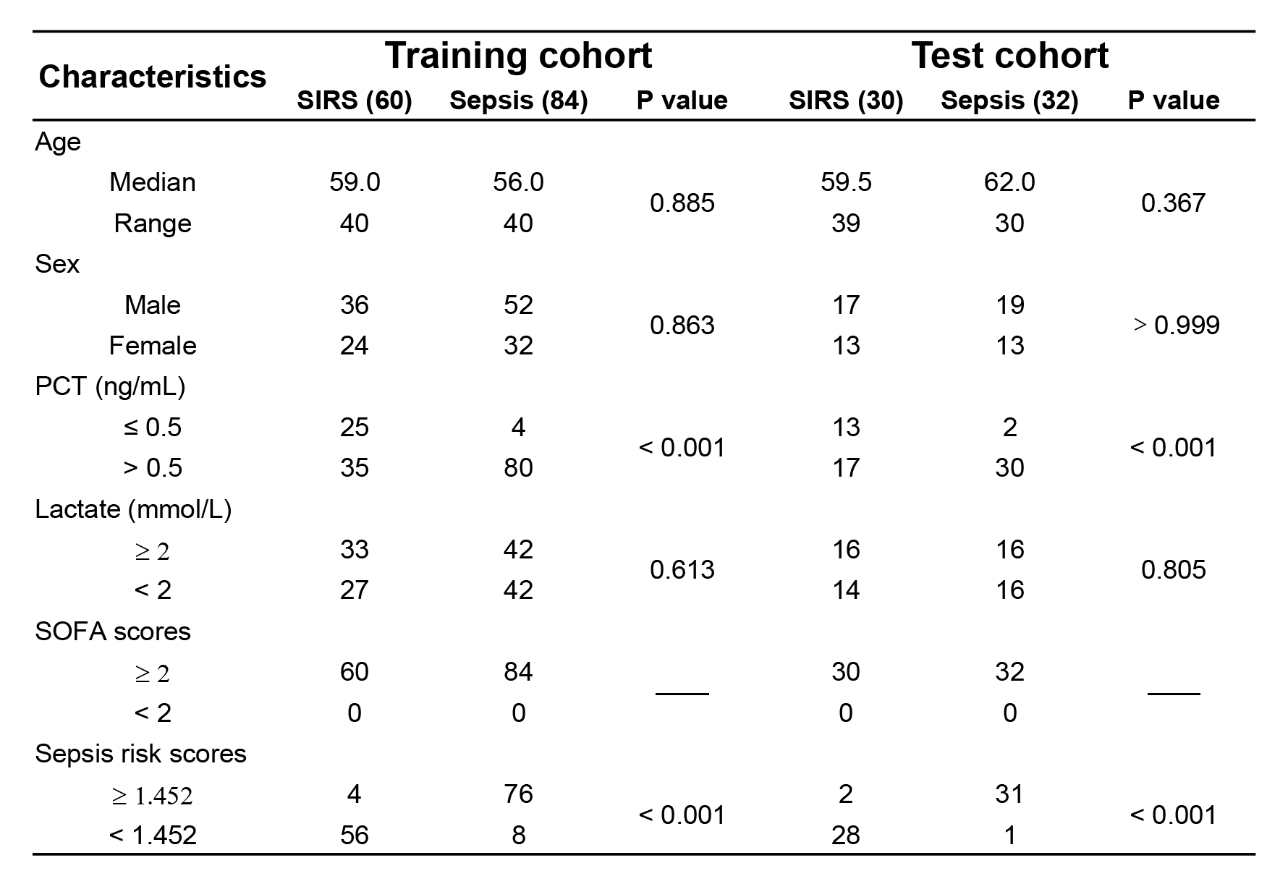
**

**Table S4.** Correlation of demographic and clinical characteristics and sepsis risk scores.

**
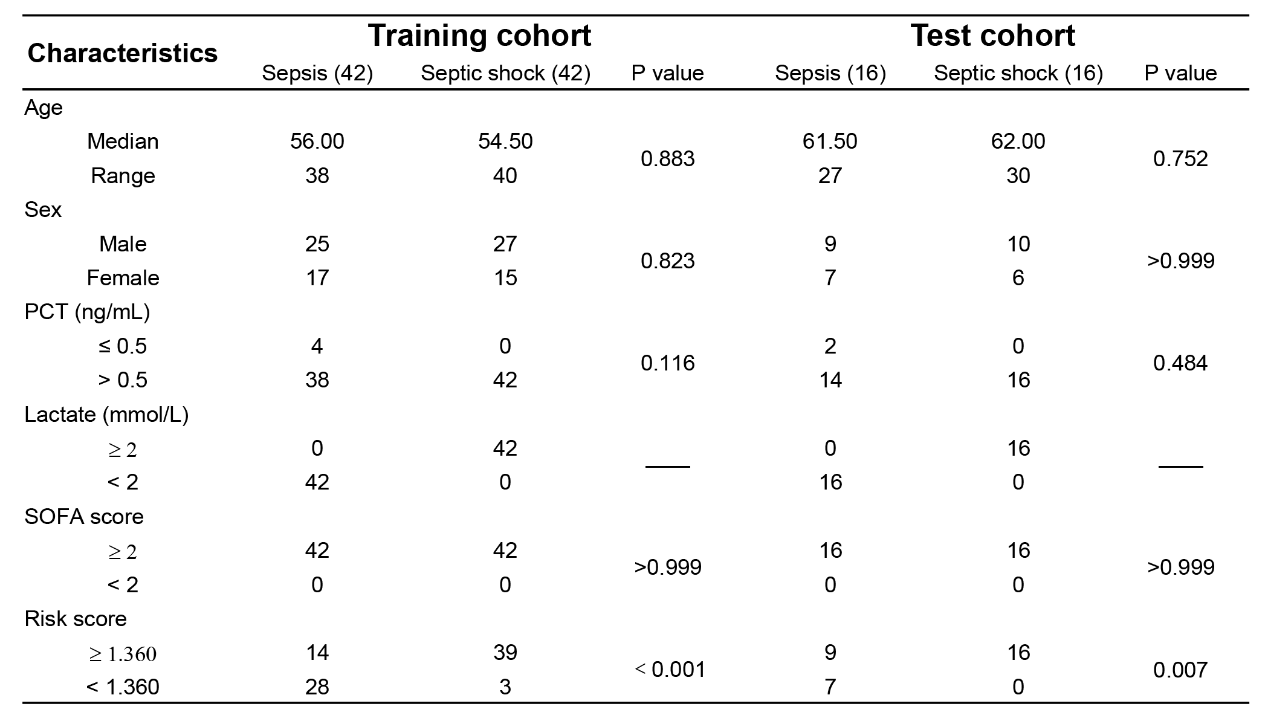
**

**Table S5.** Correlation of demographic and clinical characteristics and septic shock risk scores.


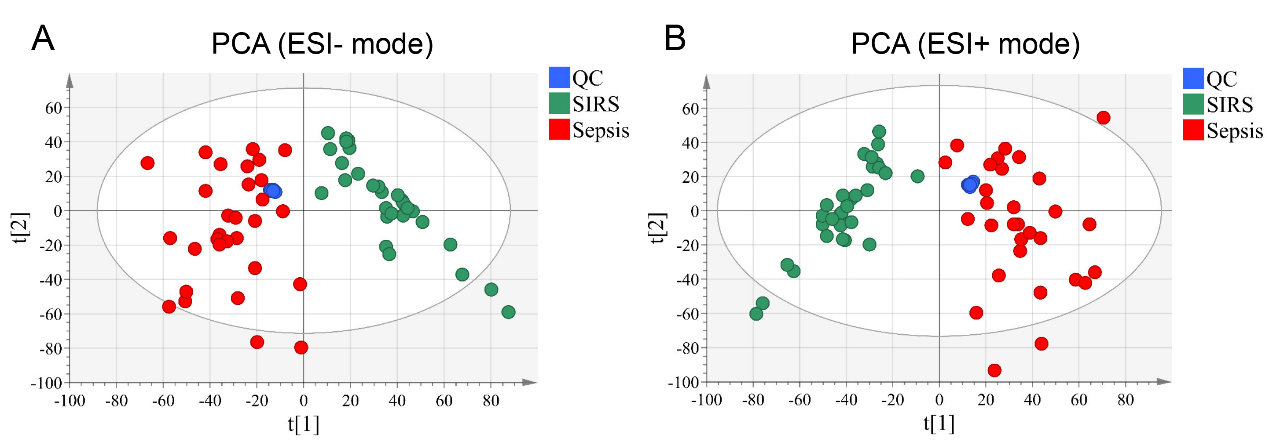


**Figure S1.** Graphical representations of PCA results obtained in negative ion mode **(A)** and positive ion mode **(B)**.


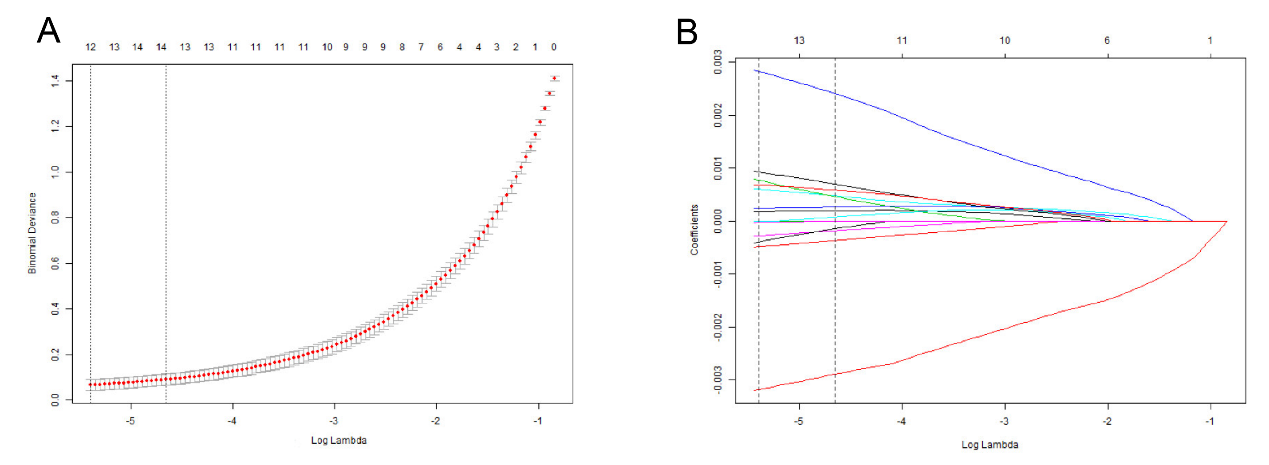


**Figure S2.** Potential metabolic biomarkers selection using the least absolute shrinkage and selection operator regularization (LASSO-LR) model. **(A)** Dotted vertical lines were drawn at the optimal values with Lambda (log), by using the minimum criteria and the 1 standard error of the minimum criteria (the 1-SE criteria). **(B)** OPLS-DA score plot of the analysis using selected metabolite.


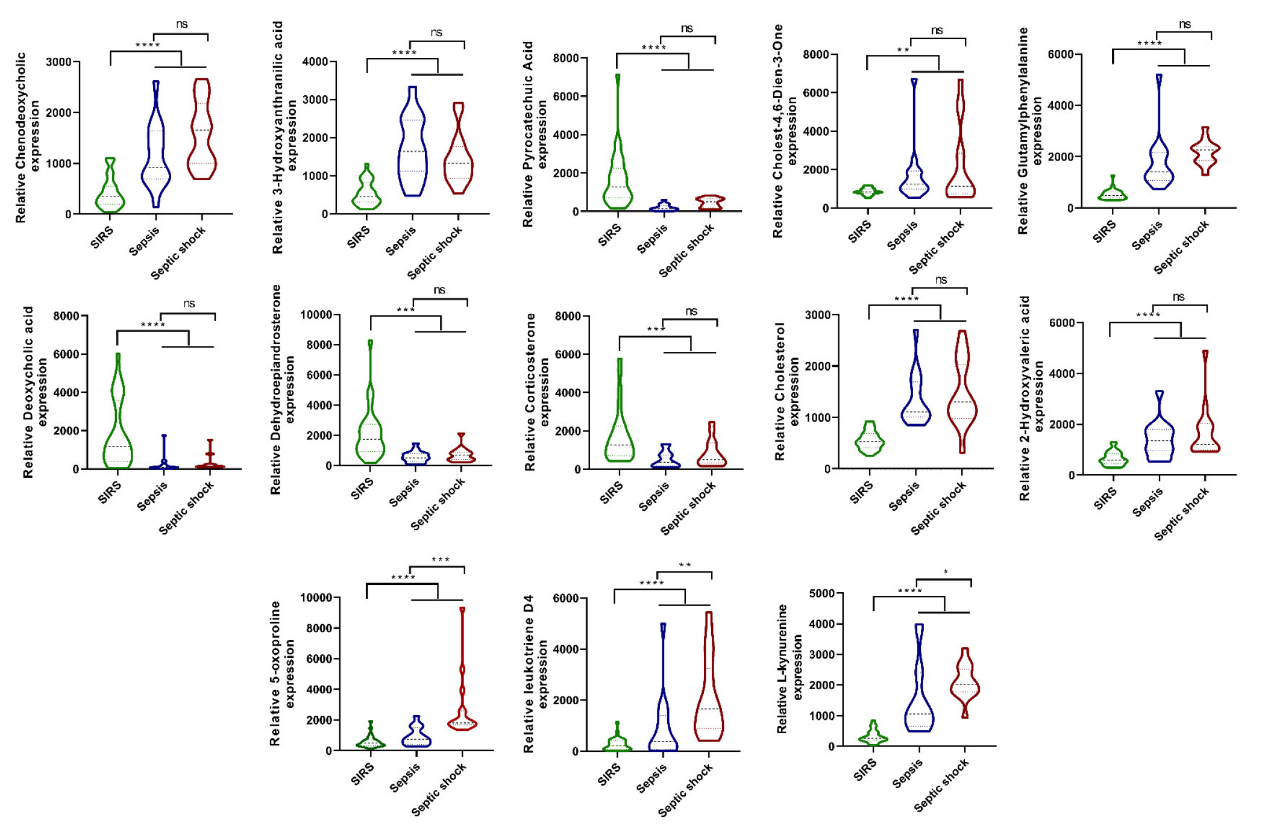


**Figure S3.** Respective expression of 5-Oxoproline, L-Kynurenine and Leukotriene D4 between sepsis and septic shock in the training and test cohorts.

**
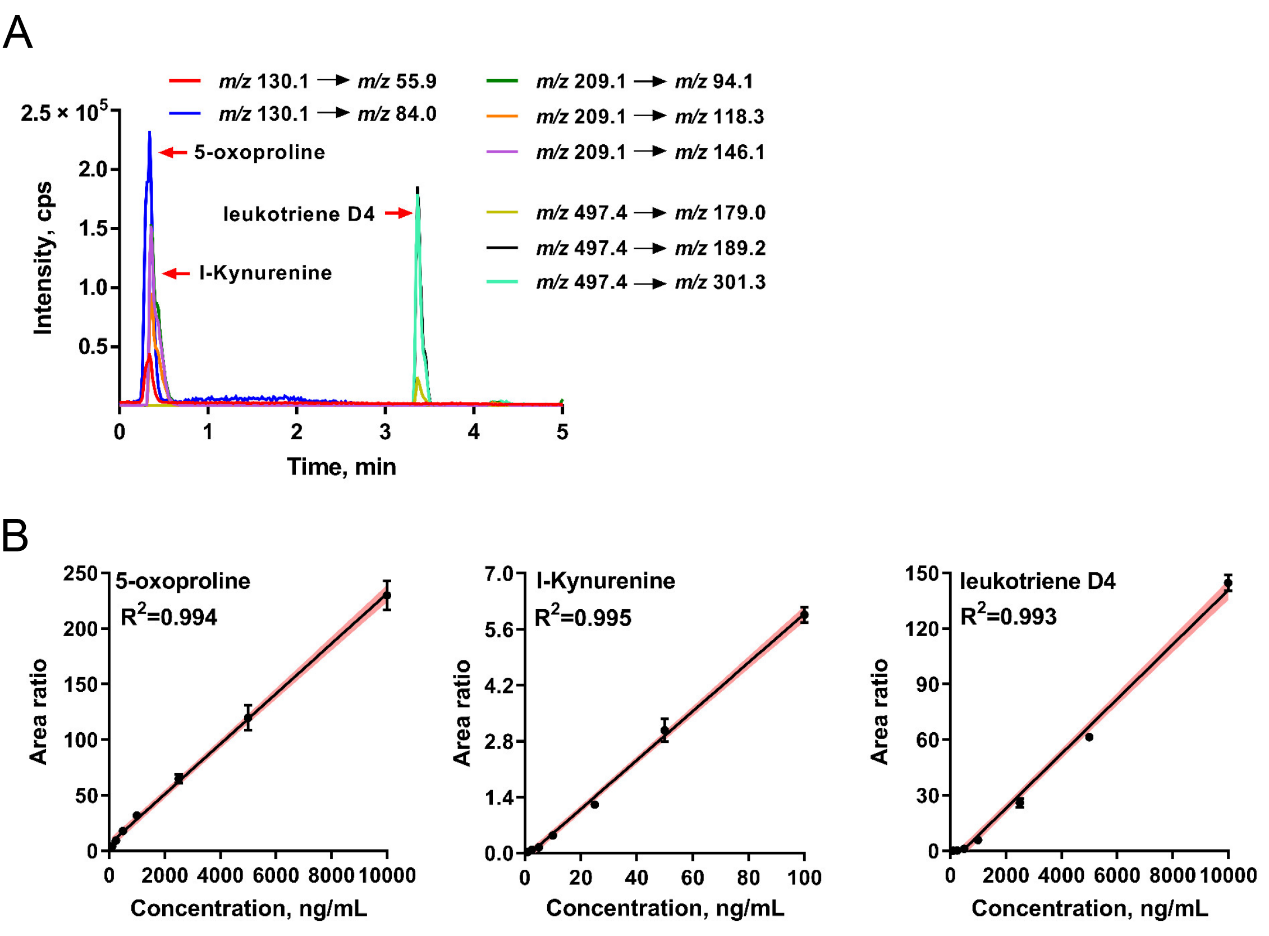
**

**Figure S4.** Development of MRM based target metabolite quantification method **(A)** and standard curve of 5-Oxoproline, L-Kynurenine and Leukotriene D4 **(B)**.

**
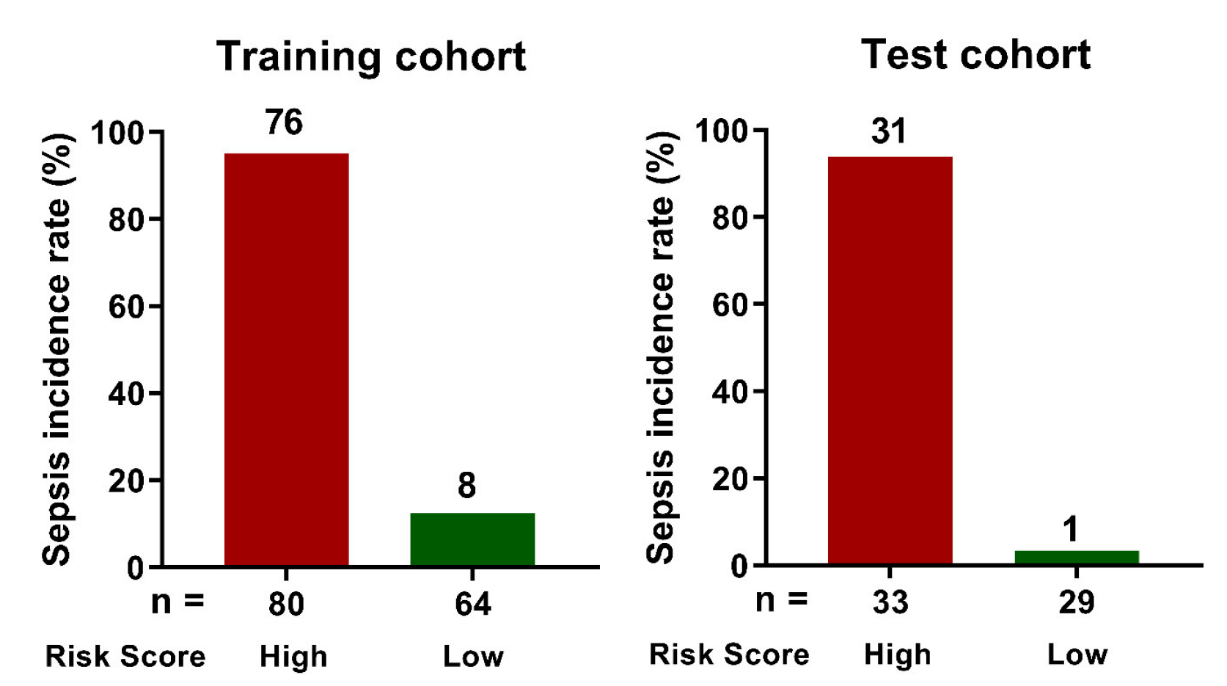
**

**Figure S5.** Investigation of sepsis incidence rate in high sepsis risk score and low sepsis risk score group which were defined based on a 3-metabolite fingerprint classifier.

**
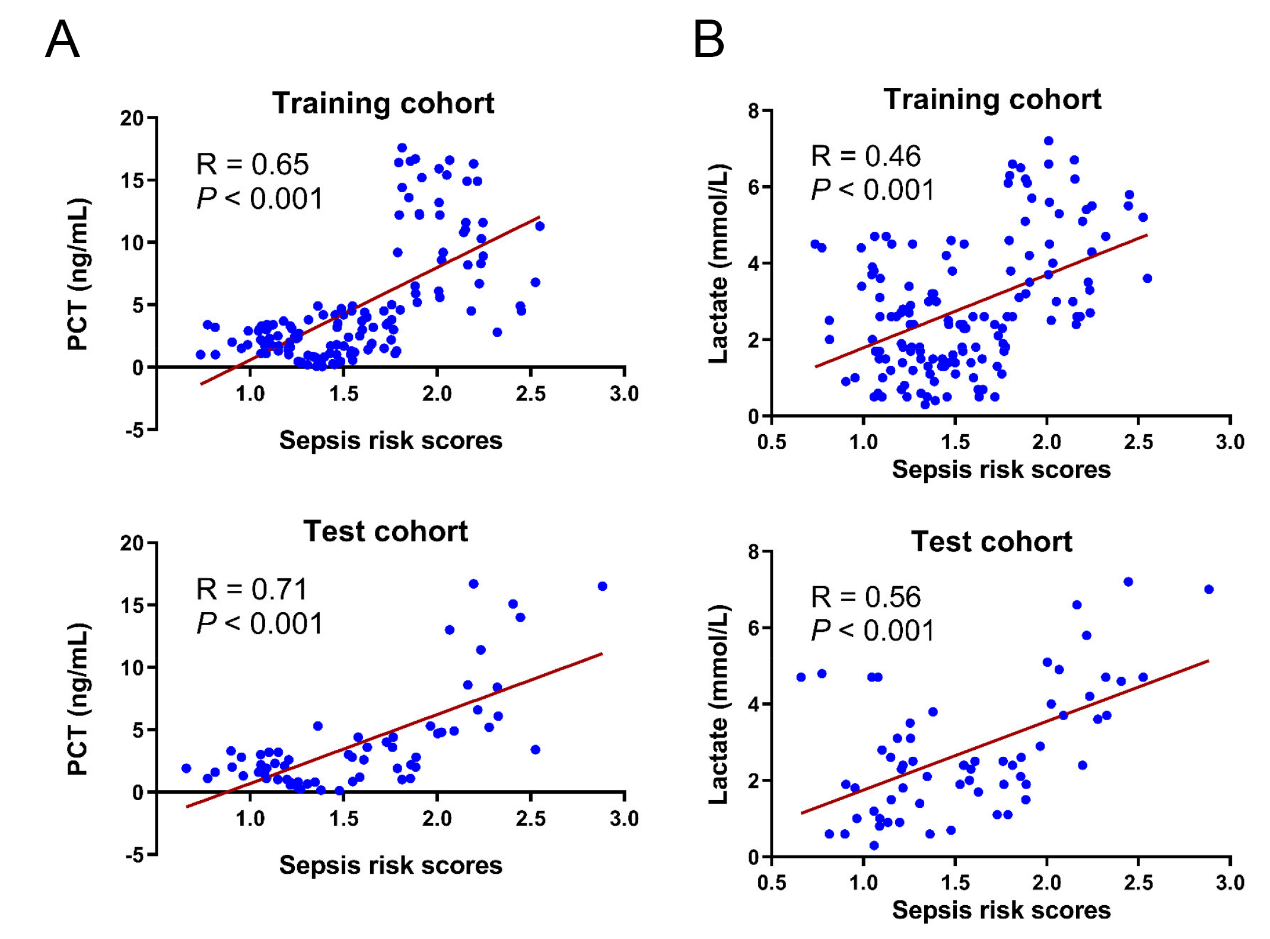
**

**Figure S6.** Correlation analysis between sepsis risk scores and PCT **(A)**, lactate **(B)**.

**
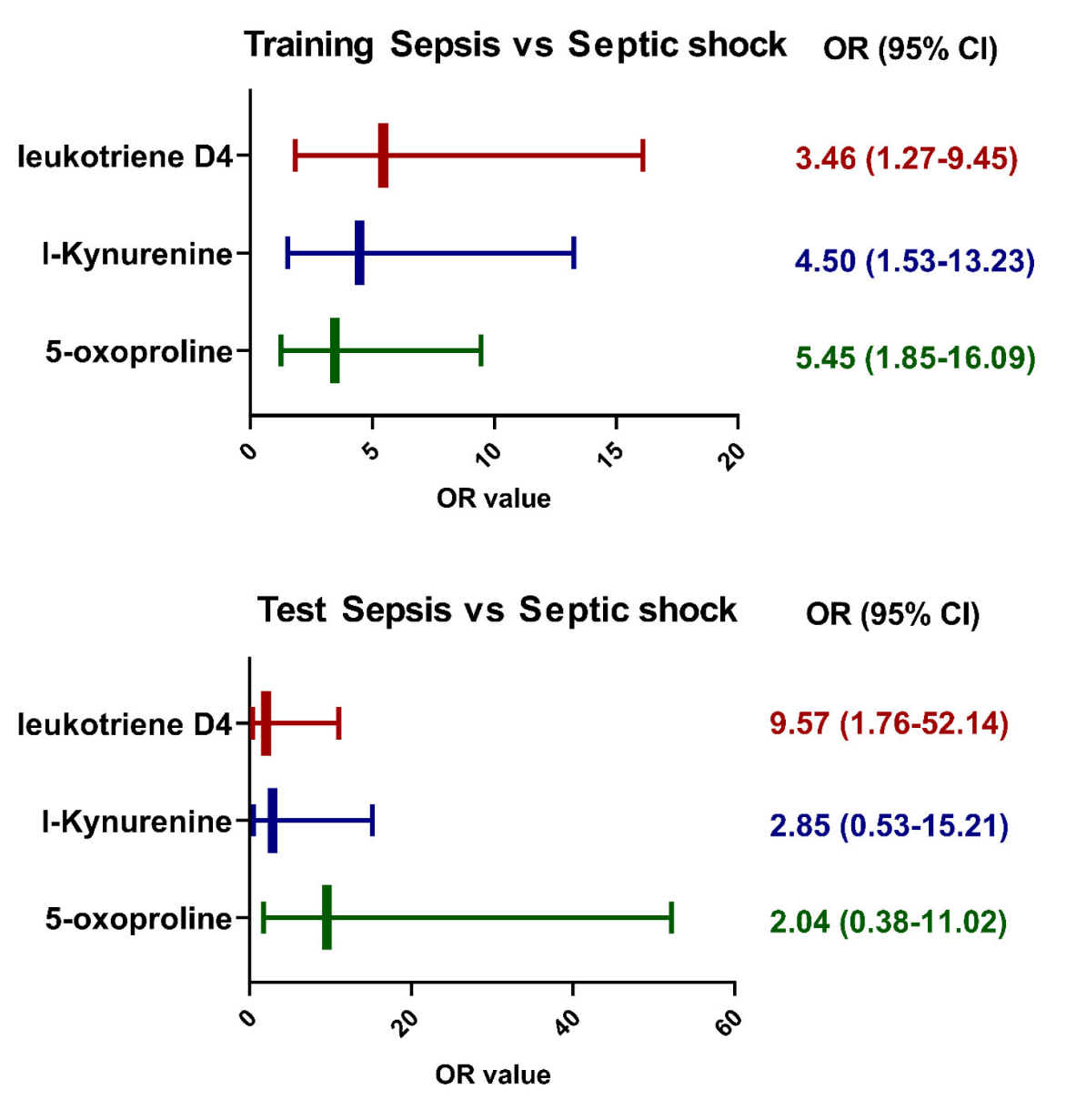
**

**Figure S7.** Odds ratios of 5-Oxoproline, L-Kynurenine and Leukotriene D4 in predicting septic shock.

**
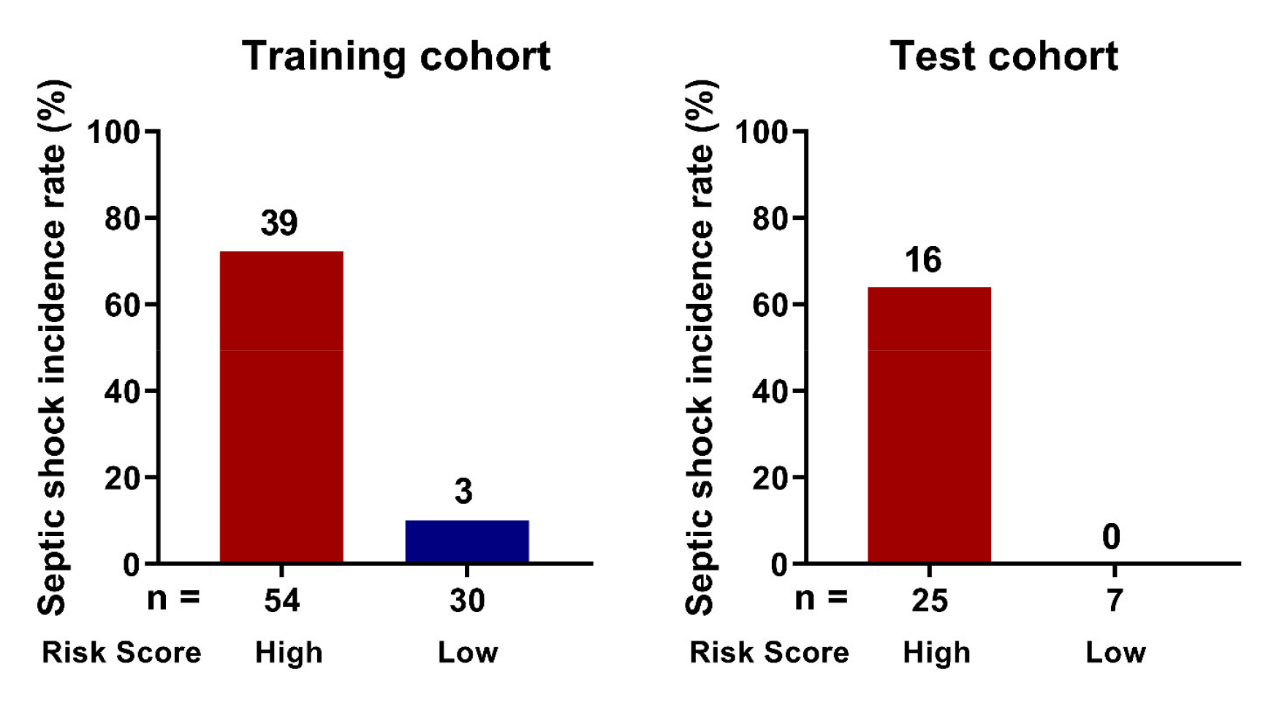
**

**Figure S8.** Investigation of septic shock incidence rate in high septic shock risk score and low septic shock risk score group which were defined based on a 3-metabolite fingerprint classifier.

**
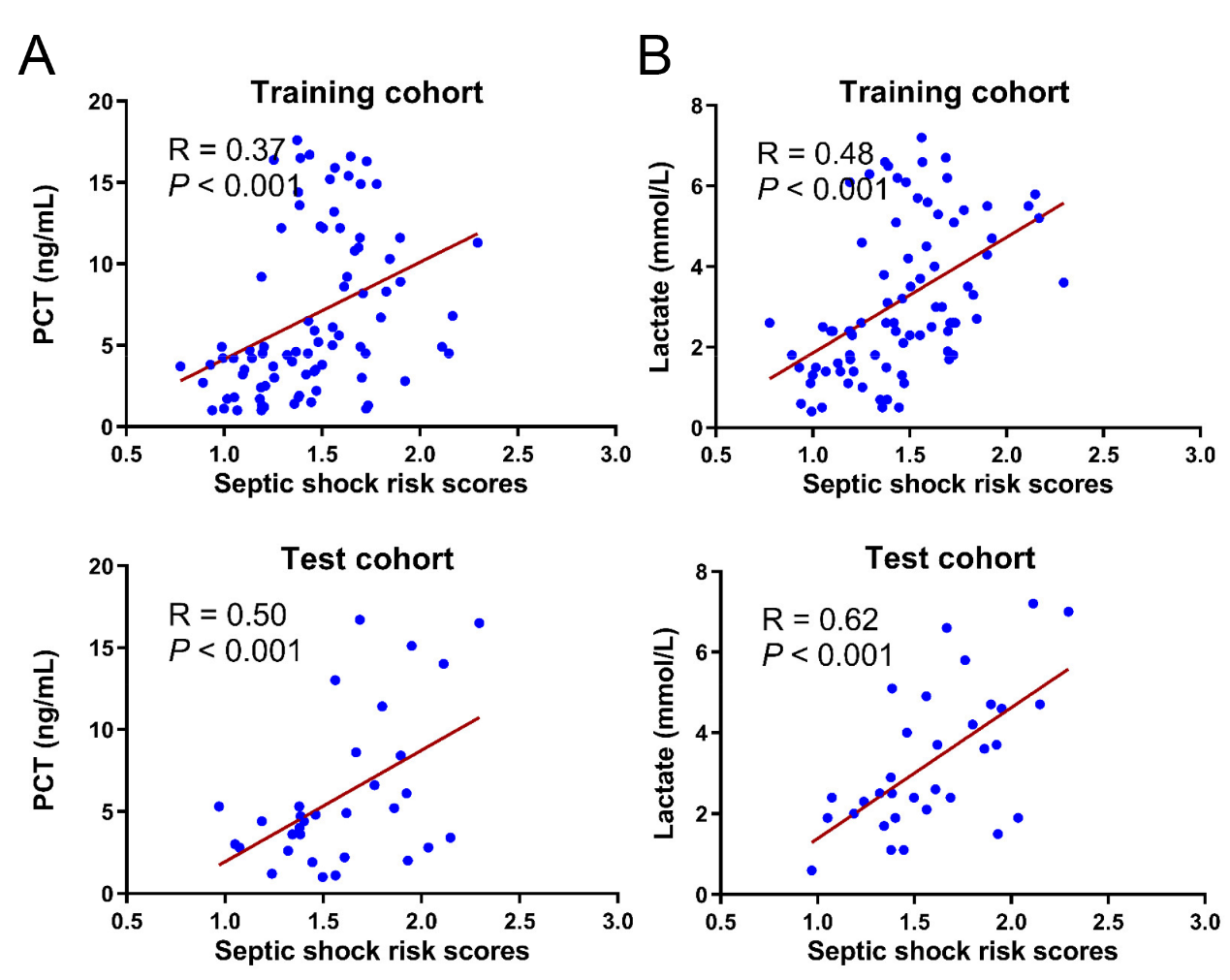
**

**Figure S9.** Correlation analysis between septic shock risk scores and PCT **(A)**, lactate **(B)**.
